# Simulating structurally variable nuclear pore complexes for microscopy

**DOI:** 10.1093/bioinformatics/btad587

**Published:** 2023-09-26

**Authors:** Maria Theiss, Jean-Karim Hériché, Craig Russell, David Helekal, Alisdair Soppitt, Jonas Ries, Jan Ellenberg, Alvis Brazma, Virginie Uhlmann

**Affiliations:** European Molecular Biology Laboratory, European Bioinformatics Institute (EMBL-EBI), Wellcome Genome Campus, Hinxton, CB10 1SD, United Kingdom; Cell Biology and Biophysics Unit, European Molecular Biology Laboratory (EMBL), Heidelberg 69117, Germany; European Molecular Biology Laboratory, European Bioinformatics Institute (EMBL-EBI), Wellcome Genome Campus, Hinxton, CB10 1SD, United Kingdom; Centre for Doctoral Training in Mathematics for Real-World Systems, University of Warwick, Coventry CV4 7AL, United Kingdom; EPSRC Centre for Doctoral Training in Modelling of Heterogeneous Systems, University of Warwick, Coventry CV4 7AL, United Kingdom; Cell Biology and Biophysics Unit, European Molecular Biology Laboratory (EMBL), Heidelberg 69117, Germany; Max Perutz Labs, University of Vienna, Department of Structural and Computational Biology, Dr.-Bohr-Gasse 9, Vienna 1030, Austria; Cell Biology and Biophysics Unit, European Molecular Biology Laboratory (EMBL), Heidelberg 69117, Germany; European Molecular Biology Laboratory, European Bioinformatics Institute (EMBL-EBI), Wellcome Genome Campus, Hinxton, CB10 1SD, United Kingdom; European Molecular Biology Laboratory, European Bioinformatics Institute (EMBL-EBI), Wellcome Genome Campus, Hinxton, CB10 1SD, United Kingdom

## Abstract

**Motivation:**

The nuclear pore complex (NPC) is the only passageway for macromolecules between nucleus and cytoplasm, and an important reference standard in microscopy: it is massive and stereotypically arranged. The average architecture of NPC proteins has been resolved with pseudoatomic precision, however observed NPC heterogeneities evidence a high degree of divergence from this average. Single-molecule localization microscopy (SMLM) images NPCs at protein-level resolution, whereupon image analysis software studies NPC variability. However, the true picture of this variability is unknown. In quantitative image analysis experiments, it is thus difficult to distinguish intrinsically high SMLM noise from variability of the underlying structure.

**Results:**

We introduce CIR4MICS (‘ceramics’, Configurable, Irregular Rings FOR MICroscopy Simulations), a pipeline that synthesizes ground truth datasets of structurally variable NPCs based on architectural models of the true NPC. Users can select one or more N- or C-terminally tagged NPC proteins, and simulate a wide range of geometric variations. We also represent the NPC as a spring-model such that arbitrary deforming forces, of user-defined magnitudes, simulate irregularly shaped variations. Further, we provide annotated reference datasets of simulated human NPCs, which facilitate a side-by-side comparison with real data. To demonstrate, we synthetically replicate a geometric analysis of real NPC radii and reveal that a range of simulated variability parameters can lead to observed results. Our simulator is therefore valuable to test the capabilities of image analysis methods, as well as to inform experimentalists about the requirements of hypothesis-driven imaging studies.

**Availability and implementation:**

Code: https://github.com/uhlmanngroup/cir4mics. Simulated data: BioStudies S-BSST1058.

## 1 Introduction

Nuclear pore complexes (NPCs) enable all macromolecular exchange between cytoplasm and nucleus. The human NPC consists of ∼30 types of proteins, called nucleoporins (Nups). Each type of Nup has a copy number of eight or multiples thereof, as Nups arrange 8-fold symmetrically around a transport axis. Nups further form three stacked rings along the nuclear envelope (NE) plane: the nucleoplasmic ring (NR) and cytoplasmic ring (CR) are called outer rings (ORs) as they sandwich an inner ring (IR) (Mosalaganti *et al.* 2022, Fig. 1, left; Supplementary Fig. S1). The abundance, size, and geometry of NPC has made it one of microscopy’s most important reference standards (Thevathasan *et al.* 2019, Sage *et al.* 2019, Pesce *et al.* 2019, Mund and Ries 2020).

**Figure 1.**
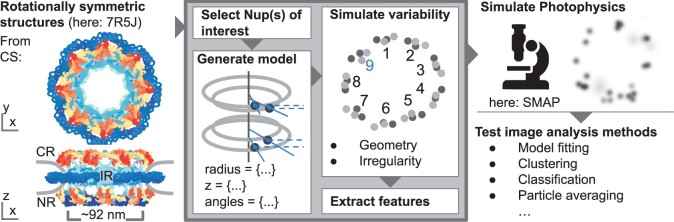
CIR4MICS workflow—our work is boxed. Left: rotationally symmetric PDB models function as input. Example Model 7R5J (Mosalaganti *et al.* 2022) was rendered using NGL viewer (Rose et al., 2018). Left, top: NPC in top-view from the cytoplasmic side (CS). Left, bottom: NPC in side-view with cytoplasmic ring (CR), inner ring (IR), and nucleoplasmic ring (NR). The nuclear envelope is shown in cut side-view. Boxed, centre: one or more Nups can be selected for labelling. Each individual Nup is encoded with a radius, angle, and *z*-position. Geometric or irregularly shaped variability can be added and ground-truth features are extracted. Right: coordinates are passed to microscopy simulation software (e.g. Ries 2020). Raw coordinates, photophysics representation, and ground truth features can be used to benchmark image analysis software.

Recent breakthroughs in *in silico* protein-structure prediction enabled near-complete models of the NPC scaffold (Jumper *et al.* 2021, Mosalaganti *et al.* 2022). However, these models achieve their pseudoatomic resolution by averaging out hundreds of NPCs and their variability (Rantos *et al.* 2022).

Examples of NPC variability are deviant symmetries (Hinshaw and Milligan 2003, Löschberger *et al.* 2014, Stanley *et al.* 2018), elliptical deformations (Beck *et al.* 2007, Allegretti *et al.* 2020, Huijben *et al.* 2021) varying azimuthal ‘twist’ angle between the ORs (Heydarian *et al.* 2021) and varying pore diameters (Stanley *et al.* 2018, Sabinina *et al.* 2021, Zimmerli *et al.* 2021, Mosalaganti *et al.* 2022). Furthermore, the distance between the ORs is inconsistently reported (Curd *et al.* 2020, Sabinina *et al.* 2021, Heydarian *et al.* 2021). Thus far, the NPC diameter has been linked to the energy status and osmotic pressure of the cell (Zimmerli *et al.* 2021, Mosalaganti *et al.* 2022) as well as to steroid-hormonal stimulation in *Xenopus laevis* oocytes (Shahin *et al.* 2005). Huijben *et al.* (2021) reported NPCs whose ellipticity aligns along a cellular axis, possibly caused by sample preparation. Depletion of lamins has been shown to aggregate NPCs near centrosomes during late G2 and prophase, possibly as lamins anchor NPCs against dynein-transport towards centrosomes (Guo and Yixian 2015). The influence of mechanical stimuli, cell cycle, signalling, and cellular energy status on NPC shape and function warrants further investigation.

Modelling approaches have been employed to functionally study NPC structural variability. Coarse-grained models and coarse grained elastic network models predicted possible deformations of the yeast NPC (Wolf and Mofrad 2008, Lezon *et al.* 2009). Molecular dynamics have been used to simulate lateral tension on the NE, which dilated a model NPC (Mosalaganti *et al.* 2022). Such theoretical models aim to predict NPC heterogeneity and are often motivated by imaging studies (Zimmerli *et al.* 2021, Mosalaganti *et al.* 2022), but conversely, can form hypotheses to be tested *ex silico*.

Single-molecule localization microscopy (SMLM) causes fluorophores to blink stochastically, thus resolving an on-state fluorophore from its off-state neighbours. SMLM can resolve Nups at a distance of 12 nm (Diekmann *et al.* 2020) and can image four differently labelled Nups in 3D (Li *et al.* 2022), thus allows to elucidate the interplay of different Nups in structurally variable NPCs. The resulting high-contrast, sparse, coordinate representation of each NPC can be fitted, classified, or averaged by downstream software (Sabinina *et al.* 2021, Huijben *et al.* 2021, Wu *et al.* 2023).

SMLM however is prone to noise, aberrations, and artefacts (Thevathasan *et al.* 2019). Consequently, it is near impossible to automatically or manually annotate structural variability of individual pores with high confidence. The ability of software to analyse NPC heterogeneity cannot be assessed without ground truth, whereas ground truth cannot be assigned without validated computational analysis. Simulated NPCs with known variability can solve this catch-22.

Wu *et al.* (2023) simulate a model of a Nup96 that can be varied in its radius, distance between the ORs, ellipticity, and twist angle between the ORs. Multiple approaches have been developed to simulate noise throughout the SMLM imaging pipeline (Venkataramani *et al.* 2016, Novák *et al.* 2017, Sage *et al.* 2019, Griffié *et al.* 2020, Ries 2020). Wu *et al.* (2023) and Ries (2020) simulate nongeometric shape variations as part of imaging noise by randomly and independently offsetting localizations from their initial positions.

These approaches however do not explicitly simulate irregular NPC shapes, i.e. shapes that are coherent, yet non-geometric, as visible in imaging studies (Löschberger *et al.* 2014, Stanley *et al.* 2018, Beton *et al.* 2021). Existing simulations furthermore integrate a very limited preselection of Nups (typically one), Nups are only loosely derived from real NPCs (Venkataramani *et al.* 2016, Huijben *et al.* 2021, Wu *et al.* 2023), and often 2D (Venkataramani *et al.* 2016, Huijben *et al.* 2021). However, explicit simulations of shape irregularity are paramount to study the robustness of fitting, classification, and averaging software to hypothesized shape irregularity. Simulations of this kind enable the benchmarking and development of methods that can distinguish between imaging noise, shape irregularity, and geometry, thereby shedding light on NPC biology.

CIR4MICS includes a computationally light-weight spring based model of the NPC to simulate geometric and irregularly shaped NPC variations. We provide a wide range of Nup models for selection which are N- and C-terminally labelled, and from human NPCs in either NE isolates or intact cells (Appen *et al.* 2015, Kosinski *et al.* 2016, Schuller *et al.* 2021, Mosalaganti *et al.* 2022). Several Nups can be chosen for multichannel simulations. Additionally, we provide datasets of geometrically variable and irregularly shaped NPCs, which includes simulated SMLM data using existing software (Ries 2020) together with our underlying ground truth coordinates and features. CIR4MICS offers a framework for benchmarking image analysis methods that are general purpose, or NPC specific, the latter of which is helpful to elucidate analysis software’s ability to capture NPC biological variability.

## 2 Materials and methods

### 2.1 Springs enable irregular but coherent deformations

Conceptually, the 8-fold symmetric NPC can be split into multiple rings containing eight Nups, where each Nup within a ring is of the same type, on the same horizontal plane, and has the same radius. Rings are henceforth called bands to avoid confusing them with subcomplexes such as the CR and NR. We simulate each band as an independent spring system to which we apply deforming forces that are spatially correlated across the NPC. This results in shape variations that are irregular due to the stochasticity of deforming forces, yet coherent as forces are spatially correlated, and as springs ensure forces are distributed throughout the band (Supplementary Fig. S4A). Strong correlation of close-by forces ensures Nup-coordinates of adjacent bands do not overlap. Springs represent the elastic and repulsive properties of proteins and membranes. Force-transmission is therefore an intrinsic properties of the model that does not have to be explicitly defined. Forces represent any cause why the NPC is not observed in its perfectly geometrical shape. Upon application of a static force, damped springs oscillate to a new static equilibrium. SMLM commonly images fixed cells. SMLM data that resolves NPC shape variability take minutes to acquire (Thevathasan *et al.* 2019, Diekmann *et al.* 2020). These data do not temporally resolve the at most second-long nucleocytoplasmic transport events (Kubitscheck and Siebrasse 2017). Consequently, we use the static new equilibrium for downstream SMLM simulations of NPCs.

### 2.2 NPC structural models function as input

Pseudoatomic structural models of the NPC exceed the resolution of SMLM and contain multiple types of Nups, facilitating multicolour simulations. SMLM usually captures NPCs in whole, but chemically fixed cells. However, models 5A9Q (Appen *et al.* 2015), 5IJN, 5IJO (Kosinski *et al.* 2016), and 7R5K (Mosalaganti *et al.* 2022) represent NPCs in isolated NEs, whereas 7PEQ, 7PER (Schuller *et al.* 2021), and 7R5J (Mosalaganti *et al.* 2022) represent NPCs in their native environment. It is therefore uncertain which models best represent NPCs under SMLM conditions. Furthermore, live-cell SMLM, in which NPCs are in their native environment, might become more common in the future (Lincoln *et al.* 2021). We therefore made all mentioned human NPC models available in our framework (Supplementary Tables S2 and S3). Our pipeline also allows for the addition of further models (Fig. 1).

### 2.3 Implementing NPC structural models

**Multichannel**: SMLM has imaged four distinct types of Nups. Each type of Nups is labelled by a distinct fluorophore (Li *et al.* 2022). We therefore enable simulations with up to six labels. Distinct types of Nups can either be treated identically or the first listed Nup type can be set as reference. Setting a reference Nup allows to compare its simulated variability between single- and multichannel simulations.

**Nomenclature**: Nups within the NPCs in top-view are arranged in *s* rotationally symmetrical ‘spokes’ around an axis of symmetry ${\vec{x}}_{c}=(x_{c},y_{c})$ (Supplementary Section S2.1). The default rotational symmetry of the NPC is s=8. ‘Rotational unit’ (RU) refers to all structurally resolved Nups in one of *s* spokes. The copy number *g* of a any selected subset of Nups within one RU corresponds to their whole-NPC copy number *m* via m=gs. They are accordingly arranged in *g* stacked bands that are parallel to the NE plane (Supplementary Fig. S1). We furthermore denote an individual Nup as *p*, and a type of Nup as *P*. Important notation is summarized in Supplementary Table S1.

**Extracting Nup coordinates from PDB**: protein termini are common fluorophore attachment sites. Thus for every mentioned PDB model, we store coordinates of the N-terminus and C-terminus of all Nups constituting a representative RU. The user can select the PDB model of interest and up to six distinct labels representing six fluorescent channels. Each label consists of a type of Nup $P_{i}$, i∈1…6 and its terminus to be tagged. Each selected fluorophore attachment site is encoded via an axial position $z_{j}$, radius $r_{j}$ from ${\vec{x}}_{c}$, and an angle relative to other fluorophore attachment sites, $\alpha_{j}$ (Supplementary Section S2.2) with j∈1…g (see Supplementary Fig. S2 for an example with two channels and g=6). The arithmetic mean $\bar{\alpha }$ of $\alpha_{j}$ is offset to 0 for single-channel simulations. For multichannel simulations, $\bar{a}=0$ if no Nup $P_{i}$ is selected as reference. Otherwise, $P_{1}=P_{\text{ref}}$ with corresponding ${\bar{a}}_{\text{ref}}$ and offset $\bar{a}$ such that ${\bar{a}}_{\text{ref}}=0$. Values are converted from Å to nm, Cartesian coordinates are generated by reflecting the selected Nups *s* times around a new central axis ${\vec{x}}_{0}$ with ${\vec{x}}_{0}=(0,0)$ and an offset of 2π/s rad. Coordinates of Nups are henceforth called ‘nodes’. A script that we used to read out coordinates from models 7R5K and 7R5J (Mosalaganti *et al.* 2022) is available and can be adapted to other structural models.

### 2.4 Layout of the spring model

**Figure 2.**
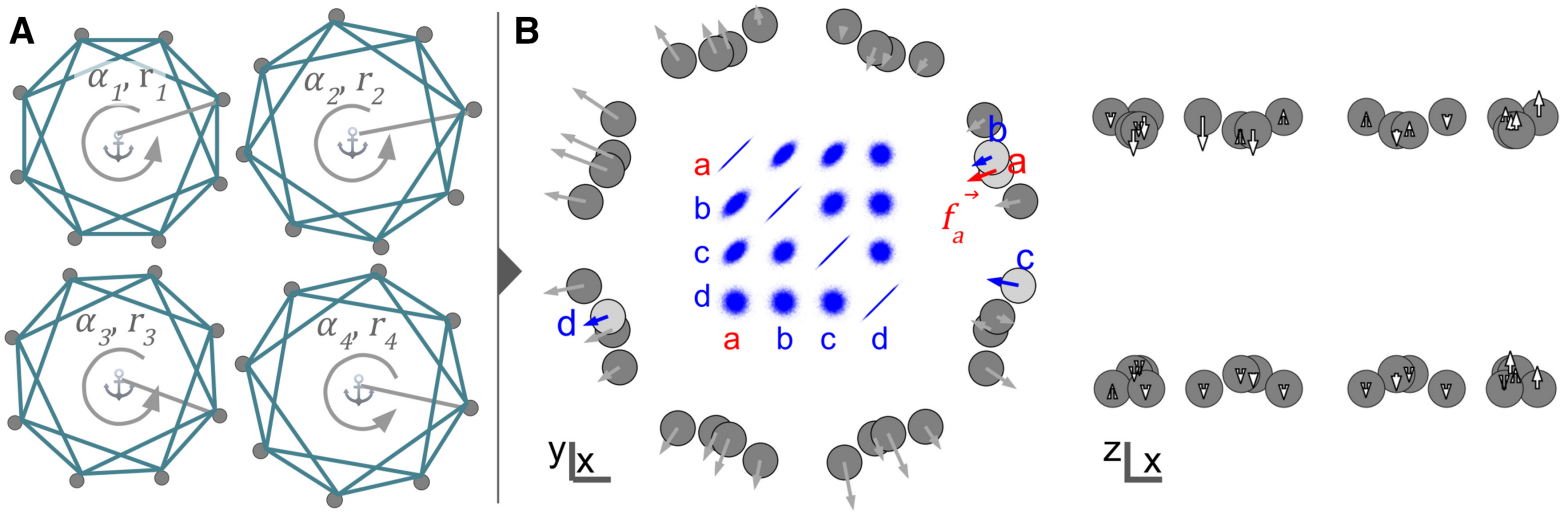
Layout of the spring model. (A) Each band *g* is simulated separately and is defined by its radius *r*, rotation α, and *z*-position (*z* not shown). (B) Continuity between independent bands is ensured with deforming forces (in *x* – *y*) or offsets (in *z*) that are covariant by distance. Left: 10 000 forces (scalar values) sampled on exemplary nodes a, b, c, and d. Right: axial offset (white arrows) are applied without springs.

We simulate each band *g* as a separate spring model rather than interconnecting the NPC at a whole-model or whole-subcomplex level. This simple, modular spring-layout ensures arbitrary numbers and types of Nups can be selected and compared (Fig. 2A) in addition to greatly accelerating computational speed. For each band, nodes are anchored to the centre via radial springs (Supplementary Fig. S3). These springs approximate the NE, whereby interior pressure replaces exterior contraction-resisting tension (Zimmerli *et al.* 2021). Nodes are connected to their 2 *h*, h∈0…⌊s/2⌋, neighbours on the same band via circumferential springs, which represent interactions within the NPC. *h* is 2 by default, as higher values increase computational cost, and lower values lead to overly jagged deformations (Supplementary Fig. S3). Shorter circumferential springs are stiffer than longer circumferential springs, indicated by their spring constants *k*. Radial springs are less stiff than the shortest circumferential spring, as we assume protein-protein interactions are stronger than protein–membrane interactions (Supplementary Section S3).

### 2.5 Stochastic forces deform the spring model laterally

Random, static forces are applied to nodes to achieve irregular shapes. Forces are sampled from a multivariate normal distribution with covariance dependent on the distance between nodes. Consequently, forces on nearby nodes are more similar than forces on distant nodes (Fig. 2B). This ensures continuity between separate NPC bands and considers that forces on real NPCs are likely not pointforces. Distances between nodes are further computed as if each band has the same arithmetic mean radius $\bar{r}$. Radial distances are therefore equalized, which preserves concentricity of bands during deformation. More precisely, covariance between forces on nodes ${\vec{x}}_{i}=(x_{i},y_{i},z_{i})$ and ${\vec{x}}_{j}=(x_{j},y_{j},z_{j})$, with i,j∈1…m are given by the RBF kernel,


(1) $$\text{cov}({\vec{x}}_{i},{\vec{x}}_{j})=e^{-\frac{{\left\|{\vec{x}}_{i}-{\vec{x}}_{j}\right\|}^{2}}{2\sigma^{2}}}$$

where ${\left\|\cdot \right\|}^{2}$ is the squared Euclidean norm, and σ is a free parameter. We couple σ to the size of the model by setting it to a fraction of the mean radius $\bar{r}$. In comparison to a linear covariance between forces and distance, the RBF kernel ensures more similarity between forces on nearby nodes, preserving local structures. This is particularly important for nearby nodes on different bands. Simultaneously, forces on distant nodes are less similar, allowing for more irregular deformations. Empirically, $\sigma =0.5\bar{r}$ produces NPC deformations that are coherent at a short scale yet overall irregular (Supplementary Fig. S4B).

**Figure 3.**
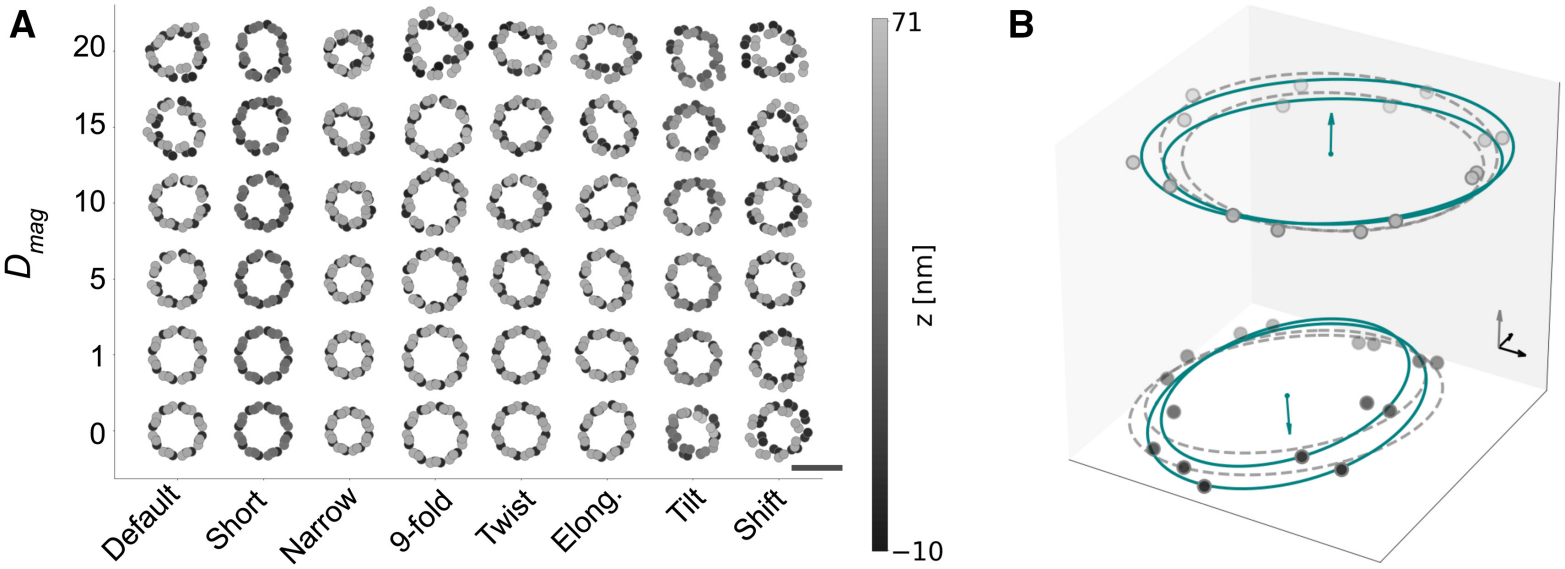
(A) Different types of geometric deformation of Nup107 at increasing $D_{\text{mag}}$ (and $O_{\text{mag}}$). Scale bar 100 nm. (B) Nup96 with $D_{\text{mag}}$ = 20. Ellipses (continuous teal line) and circles (grey dashed line) are fitted to each band. Teal arrows indicate tilt of the ORs (Supplementary Section S9.2). Grey arrow indicates *z* direction. All arrows are 10 nm long.

Lastly, the magnitude of deformation $D_{\text{mag}}$ is user-definable, so that the effects of different levels of deformation can be studied (Fig. 3A;Supplementary Fig. S4B). $D_{\text{mag}}$ [nm] covers ∼99.7 % of the sampling range [= 3 standard deviations (SD) = 3σ]. It is then converted to variance $\sigma^{\prime 2}$ via $\sigma^{\prime 2}={\left(D_{\text{mag}}/3\right)}^{2}$. Next, cov is updated as ${\text{cov}}^{\prime }=\sigma^{\prime 2}\text{cov}$ and scalar forces $f_{i}$ with i∈1…m are sampled from


(2) $$f_{i}∼N(0,{\text{cov}}^{\prime }).$$

Optionally, $f_{i}$ may be modified to include other types of deformations (see Elongation, Supplementary Section S7.6). Lastly, $f_{i}$ is transformed into a vector ${\vec{f}}_{i}=(f_{x_{i}},f_{y_{i}},0)$ that is radial and planar to avoid rotations and flips with


(3) $${\vec{f}}_{i}=(f_{x_{i}},f_{y_{i}})=\frac{f_{i}{\vec{x}}_{i}^{\prime }}{\left||{\vec{x}}_{i}^{\prime }-{\vec{x}}_{0}|\right|}.$$

${\vec{x}}_{i}^{\prime }=(x_{i},y_{i})$ thereby is the *x*–*y* position of a node to which a ${\vec{f}}_{i}$ is applied. Any set of forces with no net-torque would avoid rotations, however radial forces are simpler to compute and more intuitive. Predominantly radial forces stemming from the NE are the most parsimonious explanation for observed dilations of the NPC, particularly as no motor activity of scaffold Nups has been observed (Zimmerli *et al.* 2021, Mosalaganti *et al.* 2022). The final deformation of the model is computed for each band separately (Supplementary Section S4).

### 2.6 Random axial offset is independent of springs

Axial resolution is poorer than lateral resolution in conventional SMLM (Huang *et al.* 2008). Deforming a spring-model in only two dimensions speeds up computation and simplifies the necessary spring layout. We therefore ignore spring interactions when offsetting nodes axially. Axial offset $o_{i}$ is computed analogously to, but independently from, radial scalar forces [Equations (1–3)]. The magnitude of axial offset $O_{\text{mag}}$ is analogous to $D_{\text{mag}}$ and set to $O_{\text{mag}}=D_{\text{mag}}/2$ by default. The offset-vector on a node *i* is ${\vec{o}}_{i}=(0,0,o_{i})$. The updated node coordinates are therefore ${\vec{x}}_{i}^{\prime }={\vec{x}}_{i}+{\vec{o}}_{i}$. Following, $O_{\text{mag}}$ is implied when specifying $D_{\text{mag}}$.

### 2.7 Simulating geometric variability

Geometric variability of the NPC is commonly observed in imaging data (Hinshaw and Milligan 2003, Beck *et al.* 2007, Löschberger *et al.* 2014, Stanley *et al.* 2018, Curd *et al.* 2020, Huijben *et al.* 2021, Heydarian *et al.* 2021, Sabinina *et al.* 2021, Zimmerli *et al.* 2021, Mosalaganti *et al.* 2022). Geometric variability parameters *X* can be kept as standard input value, or redefined by the user. A measure of variation can be user-input for any parameter, except for the discrete rotational symmetry. This measure is the SD σ if not indicated otherwise. If a SD is given, a new value $X^{\prime }$ for each model NPC is drawn from a normal distribution following $X^{\prime }∼N(X,\sigma^{2}).$

When computing axial variability (Height, Tilt, Shift, Twist Angle), values are applied to the subcomplex that is closest to the cytoplasmic side (CS) and nucleoplasmic side (NS) of selected Nups, respectively. Values are interpolated for sandwiched subcomplexes. These types of variability hence do not apply if only Nups on one single subcomplex are selected. Nup coordinates do not overlap unless extreme values for geometric variability are selected.

**Height**: *d* is the difference between the average z positions of the CS (${\bar{z}}_{C}$) and NS (${\bar{z}}_{N}$) (Fig. 3A ‘Short’, Supplementary Section S7).

**Tilt**: we observed independent tilt of the CR and NR in real-data of human NPCs. Independent tilt of CS and NS can be sampled from a von Mises-Fisher distribution with parameter κ, where smaller values denote more tilt (Fig. 3A ‘Tilt’, Supplementary Section S7.3).

**Shift**: the CS and NS side can be shifted laterally, representing shear. For each side, offset in x and y-direction are independently sampled from a Gaussian distribution with mean 0 and user-defined SD (Fig. 3A ‘Shift’).

**Twist Angle**: we define the twist angle θ as the difference between the mean CS angles and the mean NS angles within one spoke (Supplementary Fig. S1, Fig. 3A ‘Twist’, and Supplementary Section S7.4).

**Radius**: the arithmetic mean horizontal distance $\bar{r}$ of Nups to the central axis. Changing the radius isotropically scales the NPC in lateral direction. Nups are therefore further apart in dilated NPCs (Fig. 3A ‘Narrow’, Supplementary Section S7.5).

**Elongation**: elongated NPCs are generated through approximation with an ellipse, whose ratio of short to long axis *q* is defined by the user. The rotational alignment between the NPC and this ellipse is randomized. Elongating forces ${\vec{f}}_{e_{i}}$ on nodes i∈1…m are sampled based on the ellipse and added to any other deforming forces (Fig. 3A ‘Elong.’, Supplementary Section S7.6).

**Symmetry**: for NPCs of varying symmetries, we keep the geodesic distance between Nups constant and preserve the absolute difference of radii between bands, which assumes the volume of a Nup is independent of its copy number. Consistently, NPCs with 9- or 10-fold rotational symmetry exhibit a greater diameter (Hinshaw and Milligan 2003, Löschberger *et al.* 2014, Stanley *et al.* 2018; Fig. 3A ‘9-fold’, Supplementary Section S7.7).

### 2.8 Interplay between geometric and irregular variability

**Figure 4.**
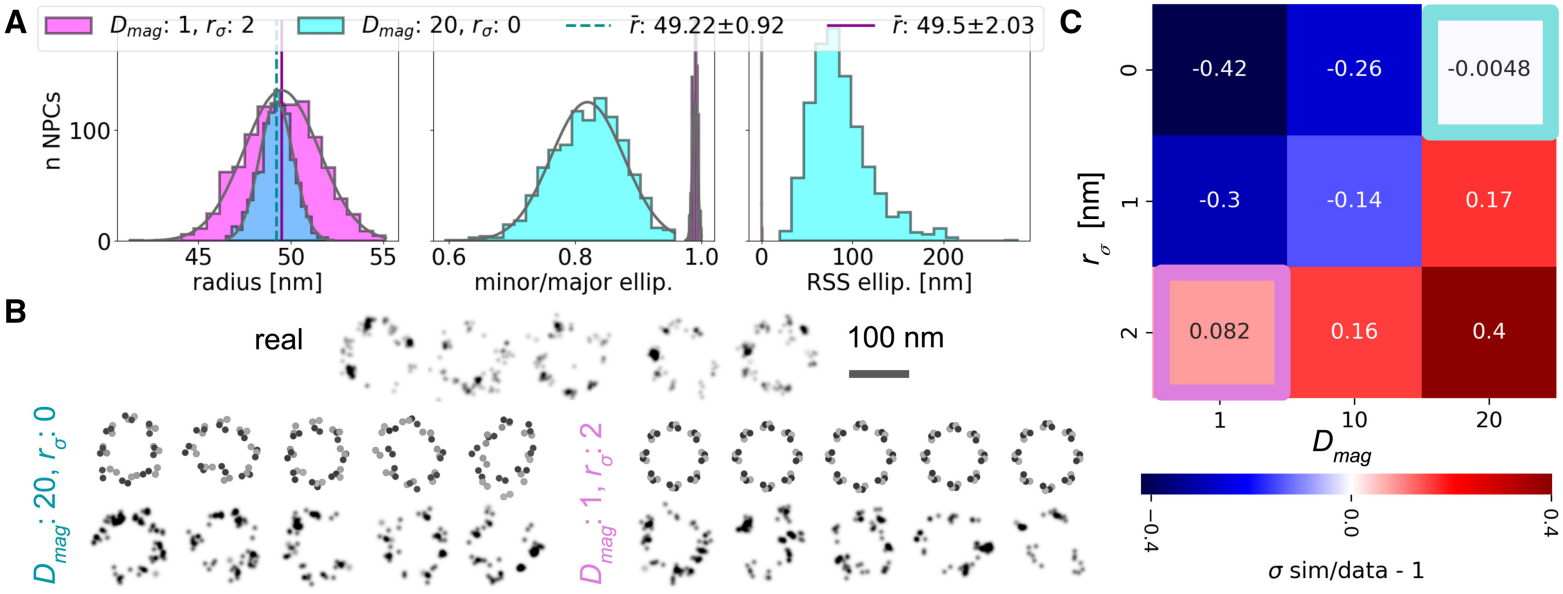
A: Distribution of extracted features of 1000 ground truth Nup96 (no photophysics) with $D_{\text{mag}}$ = 20 (magenta); and 1000 Nup96 with $D_{\text{mag}}$ = 1 and SD of radius $r_{\sigma }$ = 2 nm (teal). The radius for an individual ground truth NPCs is determined by fitting a 3D circle to each of *g* bands and averaging *g* radii. Minor/major axis ellip. and RSS ellip. is determined equivalently, but by fitting ellipses (Supplementary Section S8). Vertical lines in plot 1 indicate mean radius of ground truth NPCs. Legend indicates mean radius ± SD. (B) Example NPCs, Nup96-SNAP-AF647, Bottom left: simulations, $D_{\text{mag}}$ = 20, $r_{\sigma }$ = 0 nm, raw coordinates and with photophysics. Bottom right: simulations, $D_{\text{mag}}$ = 1, $r_{\sigma }$ = 2 nm, raw coordinates and with photophysics. Top: corresponding real NPCs from Thevathasan *et al.* (2019). (C) Nup96-SNAP-AF647, Comparison of radial SD between simulated data (with photophysics) and real data. Values >0 (red-shift) indicate radial SD of simulated NPCs is higher. 1000 NPCs per combination of $r_{\sigma }$ and $D_{\text{mag}}$. Real data: $r_{\sigma }$ = 2.1 nm, real value and analysis pipeline from Thevathasan *et al.* (2019). Magenta and teal boxes highlight example parameters shown in (A) and (B).

Irregular forces and correlated axial offset will deform the NPC model from its input geometric parameters (Fig. 4A). Irregular variability can thus be disentangled into a geometric component and true irregularity, which represents irregular shapes visible in imaging data (Löschberger *et al.*, 2014, Stanley et al. 2018, Beton *et al.* 2021). Our framework hence allows to tune the proportion of true irregularity, as well as the relative proportions of different types of geometric variability.

### 2.9 Extracting features

Features are computed using parameters of fitted structures: we fit a 3D circle and 3D ellipse to each sub-complex, or to each band *g* (Fig. 3B). We define subcomplexes as CR, IR, NR, the bridge between CR and IR (BRCR), and the bridge between IR and NR (BRNR) (Supplementary Table S3 and Sections S8 and S9).

### 2.10 Output files and image data simulation

Labelling efficiency, i.e. the ratio of successful labels to fluorophore binding sites, can be user-input in case downstream simulation software do not offer this functionality. An expansion factor can be set to simulate expansion microscopy. For each simulation experiment, NPC coordinates are saved in a CSV file that can be loaded into photophysics simulation software (e.g. Ries 2020). A metadata file with properties of simulated NPCs is generated, as well as a CSV file containing features of circles and ellipses fitted to subcomplexes (CR, BRCR, IR, BRNR, and NR), a CSV file with circle- and ellipse features fitted to individual bands, and a CSV file containing circle-and ellipse features per NPC obtained by averaging *g* band-features (Supplementary Section S10).

## 3 Results

### 3.1 Simulated datasets

We used PDB model 5A9Q (Appen *et al.* 2015) as basis for simulations due to its high pseudoatomic resolution. We selected N-terminally labelled Nup107 (short: Nup107) and C-terminally labelled Nup96 (short: Nup96), (Supplementary Tables S4 and S3), which are frequently studied (e.g. Thevathasan *et al.* 2019, Sabinina *et al.* 2021, Li *et al.* 2022, Wu *et al.* 2023). We used Nup96 to recapture two specific imaging experiments carried out in Thevathasan *et al.* (2019): good-quality imaging with the tag SNAP-AF647, and poorer-quality with the tag mMaple. A virtual nucleus with 1000 strongly deformed NPCs (Nup96) takes 45–51 s to simulate and export on a personal laptop (Ubuntu Linux 20.04, processor model: i7-8550U, Processor frequency: 1.80 GHz, and RAM: 16 GB).

**N-Nup107**: we simulated eight classes representing commonly observed or hypothesized variability: unmodified reference NPCs, smaller radius, smaller CR-NR distance, elongated NPCs, 14${}^{{}^{\circ}}$ twist angle, 9-fold symmetry, shifted CR and NR, and independently tilted NR and CR (Supplementary Table S5; Fig. 3A). For each class, we simulated NPCs with $D_{\text{mag}}$ 0, 1, 5, 10, and 15; 1000 NPCs each, and generated output files according to Methods ‘Output files and Image simulation’.

**Nup96-C**: Thevathasan *et al.* (2019) published a simple geometric analysis of Nup96 radii based on well-characterized imaging data. We simulated comparable ground-truth NPCs by sweeping two parameters that contribute to the variability of fitted NPC radii: The SD of NPC radius $r_{\sigma }$, and irregular variability $D_{\text{mag}}$ (Supplementary Table S6). $D_{\text{mag}}$ contributes to geometric variability, such as ellipticity, tilt, height, and shift, as well as to true irregular variability, measured as the residual sum of squares (RSS) from band-wise, 3D-fitted ellipses (Fig. 4A;Supplementary Fig. S7 and Section S9). The standard-deviation of radii $r_{\sigma }$ fitted to 2D NPCs in real-data was 2.1 nm for good-quality Nup96-SNAP-AF647 and 3.5 nm for worse-quality Nup96-mMaple (Thevathasan *et al.* 2019). We therefore simulate each 1000 NPCs with $r_{\sigma ,\text{model}}$ of 0, 1, and 2 nm; as well as $D_{\text{mag}}$ of 1, 10, and 20, resulting in 9 combinations of $r_{\sigma ,\text{model}}$ and $D_{\text{mag}}$ (Fig. 4B and C).

### 3.2 Simulated image acquisition

Photophysics were simulated in SMAP (Ries 2020). Photophysics parameters were modified from Wu *et al.* (2023), which represents a good-quality imaging experiment. Wu *et al.* (2023) simulate irregularity as a random, independent offset from the expected localization position called ‘free linkage error’. Because our model accounts for shape irregularity with $D_{\text{mag}}$, we are able to reduce the free linkage error to what it is expected to be in reality, in this case the size of the SNAP-tag (Yao *et al.* 2021) or mMaple (Supplementary Table S9). For Nup107, simulation parameters are shown in Supplementary Table S7. For Nup96, we further modify photophysics-parameters to correspond to Thevathasan *et al.* (2019) (Supplementary Tables S8 and S9). Simulated imaging data were exported as CSV files.

### 3.3 Comparison with real NPCs

In our pipeline, parameters of simulated geometric, and irregular shape variability can be adjusted, and features of resulting NPC epitopes can be directly read-out. This allows to systematically disentangle factors contributing to NPC variability. In combination with photophysics-simulations, this offers a reference to variability in real-data. We ran the radius-analysis from Thevathasan *et al.* (2019) on our simulations. $r_{\sigma ,\text{model}}$ and $D_{\text{mag}}$ have little influence (<0.5 nm) on the arithmetic mean radius $\bar{r}$ per dataset, even with added photophysics (Fig. 4A;Supplementary Fig. S6). We therefore compared the SD of radii $r_{\sigma }$ of real and simulated datasets with


(4) $$\frac{r_{\sigma ,\text{sim}}}{r_{\sigma ,\text{real}}}-1,$$

such that negative values indicate too little simulated variability, and positive value too much simulated variability.

The radius-variability of real NPCs can both be achieved by predominantly varying the underlying model-radius, as well as by only simulating irregular variability (Fig. 4B and C). Assuming that the simulated photo-physics accurately capture real-life photophysics, we can exclude combinations of $r_{\sigma ,\text{model}}$ and $D_{\text{mag}}$ that have too much or too little radial variability. Simulated good-quality image data (Nup96-SNAP-AF647, Fig. 4C) is consistent with simulated poorer-quality image data (Nup96-mMaple, Supplementary Fig. S7) in that eight of the nine simulation experiments agree on whether the simulated SD is too high Eq. 4> 0 or too low Eq. 4<0. One experiment ($r_{\sigma }=2,D_{\text{mag}}=1$, magenta box) differs in its sign, but both values are close to 0. Nup96-mMaple overall exhibits values closer to 0, suggesting a greater relative contribution of imaging noise to sample variability.

## 4 Discussion

Current SMLM simulation methods cover the entire imaging pipeline including basic variations of NPC geometry (Ries 2020 Huijben *et al.* 2021, Wu *et al.* 2023). However, no approach allows simulating irregular but coherent shape variations. Here, we introduce CIR4MICS, a toolbox that simulates irregularly shaped NPCs as well as a wide range of geometric NPC variability reported in literature and anecdotally. We provide a reference dataset that includes deformed ground truth NPCs, annotated features, as well as corresponding simulated imaging data (Ries 2020). Amongst other applications, our software allows to compare fluorescence microscopy clustering, classification, and averaging methods. A side-by-side comparison with real data allows to find the simulation parameters that best represent real NPCs, thus elucidating their structural variability.

We integrated various pseudoatomic models of human NPCs into CIR4MICS (Appen *et al.* 2015, Kosinski *et al.* 2016, Schuller *et al.* 2021, Mosalaganti *et al.* 2022) and provide a tutorial and scripts on how to integrate further models. Simulations can therefore be extended to NPCs of other species such as yeast (Kim *et al.* 2018, Allegretti *et al.* 2020, Zimmerli *et al.* 2021), especially since advances in integrative modelling (Rantos *et al.* 2022, Mosalaganti *et al.* 2022) promise a wider gamut of resolved NPCs. Generally, CIR4MICS allows to integrate any rotationally symmetric structure, such as cilial cross-sections, with little to no modifications (Shi *et al.* 2019). Filamentous structures could further be simulated by increasing the model symmetry such that local topology resembles a line segment.

In our framework, Nup coordinates are transformed into a spring model to which static deforming forces are applied to achieve irregular, yet coherent shapes. Our simulations resemble coarse grained elastic models (Wolf and Mofrad 2008, Lezon *et al.* 2009) or molecular dynamics simulations (Mosalaganti *et al.* 2022) in that a model is deformed by forces. However, we do not intend to predict true NPC structural variability, but to replicate observed or presumed variability as synthetic ground truth. Such hypothesis-driven approaches require multiparameter sweeps and a rapid turnover of updated hypotheses and simulations, thus benefit from fast computations. CIR4MICS’ simple model layout allows to generate a virtual nucleus of 1000 annotated NPCs in under one minute on a standard laptop.

Our framework has already been beneficial in a previous study, where we examined the behaviour of two classification methods on distinct geometrical classes (Theiss 2022). Particle averaging methods align degraded observations to reconstruct their underlying structure, but struggle with biological heterogeneity. Particles can therefore be clustered prior to averaging (Huijben *et al.* 2021, Sabinina *et al.* 2021, Theiss 2022), or deformed into a common underlying shape (Shi *et al.* 2019). Our framework can be used to study how increasing levels of shape irregularity affect clustering, classification, and averaging. Furthermore, relative distances of localizations of multiple structures can be statistically analysed to extract information of the underlying average geometry (Curd *et al.* 2020). CIR4MICS allows to study the effect of biological variability on such analyses.

Geometric model fitting characterizes underlying structures in terms of geometric parameters, which can form the basis for clustering, classification, and particle averaging (Thevathasan *et al.*, 2019, Wu *et al.* 2023). However, model-structure mismatch affects parameter fitting reliability (Wu *et al.* 2023). We elaborate on this notion of mismatch by simulating biological shape irregularity as parameter $D_{\text{mag}}$. Parameter $D_{\text{mag}}$ contributes to geometric variability, as well as to true irregularity, such as the RSS from an optimally fitting geometric representation. By adjusting $D_{\text{mag}}$ and geometric parameters, we can finetune the proportions of distinct types of variability in a dataset. Reproducing a simple circle-fitting geometric analysis (Thevathasan *et al.* 2019) on simulated data, we showed that both predominantly geometric variability and predominantly irregular variability can replicate variability of radii found in real data. Previous simulations do not allow to distinguish between true radial variability and coherent shape irregularity (Venkataramani *et al.* 2016, Huijben *et al.* 2021, Wu *et al.* 2023). Assuming equal imaging noise, we can conclude that a combination of biological variability parameters that exceeds or undershoots variability of real data, does not represent real NPCs.

The here discussed analysis assumes an unimodal distribution of NPC radii. However, the distribution underlying geometric parameters of real NPCs is unknown. For example, NPCs are commonly 8-fold symmetric, however, 9- and 10-fold symmetric NPCs have been reported (Hinshaw and Milligan 2003, Löschberger *et al.* 2014, Stanley *et al.* 2018). Other aspects of NPC geometry might be truly unimodal; or multimodal, but not detectable by analysis software; or unimodal with classification methods falsely reporting multimodality. Crucially, CIR4MICS can synthesize ground truth to assess the amount and quality of data needed to answer a particular question using a given software. These synthetic data hence aid in the choice of analysis methods, help develop new methods, and inform experimentalists about the feasibility and requirements of a given analysis. Users can for instance use the pre-generated two-class dataset, say NPCs with twist-angle of 9.6${}^{{}^{\circ}}$ and 14${}^{{}^{\circ}}$ (Heydarian *et al.* 2021), to test whether an analysis method recovers this bi-modality. Results can be compared to equivalent analyses on real-NPCs. Users can further sweep parameters to test the points where bi-modality is no longer recoverable: The difference between the modes can be varied, and angles can be drawn from a Gaussian distribution with increasing SD. The shape irregularity $D_{\text{mag}}$ can be increased and the number of simulated NPCs per class can be adjusted to find the minimum number required for statistical significance.

CIR4MICS is not limited to SMLM simulations, as several microscopy techniques work on the basis of attaching fluorescent or electron-dense labels to a structure. The label coordinates generated by our method can therefore be passed to simulation pipelines of other microscopy techniques. Our framework allows for reduced labelling efficiency and the addition of an expansion factor, thus enabling the simulation of expansion microscopy, for instance in combination with structured illumination microscopy (Ahmad *et al.* 2021). More generally, CIR4MICS combines molecular physics simulations with microscopy simulations. Such interfaces will enable realistic image simulations and a direct comparison of structural simulations with imaging data.

## Supplementary Material

btad587_Supplementary_Data

## Data Availability

All described codes are available at https://github.com/uhlmanngroup/cir4mics. Simulated data are available at BioStudies (Accession number S-BSST1058).
